# Archaeopedological analysis of colluvial deposits in favourable and unfavourable areas: reconstruction of land use dynamics in SW Germany

**DOI:** 10.1098/rsos.171624

**Published:** 2018-05-30

**Authors:** Jessica Henkner, Jan Ahlrichs, Sean Downey, Markus Fuchs, Bruce James, Andrea Junge, Thomas Knopf, Thomas Scholten, Peter Kühn

**Affiliations:** 1 SFB1070 ResourceCultures, Gartenstr. 29, 72074 Tübingen, Germany; 2 Department of Geosciences, Soil Science and Geomorphology, Eberhard Karls University Tübingen, Rümelinstrasse 19-23, 72070 Tübingen, Germany; 3 Institute for Pre- and Protohistory and Medieval Archaeology, Eberhard Karls University Tübingen, Burgsteige 11, Schloss Hohentübingen, 72070 Tübingen, Germany; 4 Department of Anthropology, The Ohio State University, 4034 Smith Laboratory, 174 West 18th Street, Columbus, OH, 43210, USA; 5 Department of Geography, Justus-Liebig-University Gießen, Senckenbergstr. 1, 35390 Gießen, Germany; 6 Department of Environmental Science and Technology, University of Maryland, 0220 Symons Hall, College Park, MD 20742, USA

**Keywords:** colluvium, past soil erosion, dating, human impact, soil, OSL

## Abstract

Colluvial deposits, as the correlate sediments of human-induced soil erosion, depict an excellent archive of land use and landscape history as indicators of human–environment interactions. This study establishes a chronostratigraphy of colluvial deposits and reconstructs past land use dynamics in the Swabian Jura, the Baar and the Black Forest in SW Germany. In the agriculturally favourable Baar area multiple main phases of colluvial deposition, and thus intensified land use, can be identified from the Neolithic to the Modern times. In the unfavourable Swabian Jura increased colluvial deposition began later compared to the more favourable areas in the Baar. The same holds true for the unfavourable areas of the Black Forest, but intensified land use can only be reconstructed for the Middle Ages and Early Modern times instead of for the Bronze and Iron Age as in the Swabian Jura. Land use intensity and settlement dynamics represented by thick, multilayered colluvial deposits increase in the Baar and the Black Forest during the Middle Ages. In between those phases of geomorphodynamic activity and colluviation, stable phases occur, interpreted as phases with sustainable land use or without human presence.

## 1. Introduction

The spread of agriculture through central Europe, starting about 7500 years ago, changed food production and population densities [1]. The establishment of sedentary lifestyles resulted in increased and diversified food production for subsistence purposes and also production of surpluses for sale, labour specialization and social complexity [2]. It also led to increased alteration of the natural landscape [3,4]. This crucial change of the relationship between humans and the environment can be traced with archaeopedological methods. Thus, soils are records of past land use [5,6] and they are resources to learn about past human–environment interactions. Archaeopedology is defined as the study of site formation history, cultural chronology, land use (change) and environmental change; and it allows us to answer archaeological questions with pedological methods [7–9]. In our study, archaeopedological methods include soil description, chemical and physical soil analyses, and dating of colluvial deposits and charcoal fragments. The used methods do not allow to infer the type of land use and thus the term land use comprises all forms of human land use such as deforestation, mining, village establishment or infrastructure building, and farming (i.e. cultivation and animal husbandry). The latter marks the beginning of a more intense and permanent anthropogenic land use, which led to widespread formation of colluvial deposits. Hunter and gatherer populations also used the land and depended on the soil, but non-sedentary societies had smaller impacts on soil erosion and colluvial deposition than agricultural societies [10]. Colluvial deposits are the correlating sediments of human-induced soil erosion and as such their distribution is mainly controlled by topography, precipitation and human activities [11]. Larsen *et al*. [12] state that land use, rather than soil erosion rates and discharge into the oceans, controls temporary sediment storage on slopes, which means the analysis of colluvial deposits on slopes and in depressions is ideal to reconstruct land use change.

**Figure 1.**
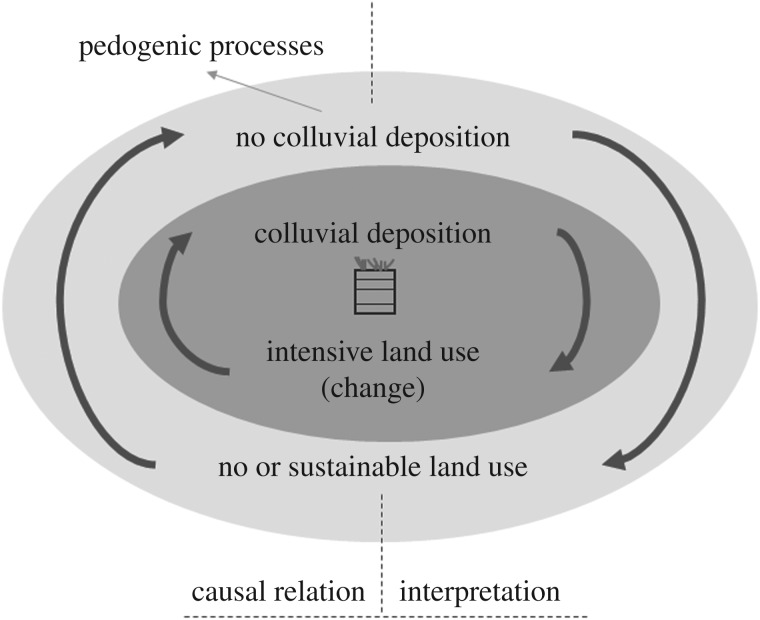
Schematic diagram of the interpretation and causal relationship between colluvial deposition and land use. Dark grey, geomorphodynamic activity; light grey, geomorphodynamic stability.

The use of colluvial deposits as records of the past is based on the assumption that intensified land use, as caused by agriculture, results in soil erosion and temporary storage of sediments along slopes or in depressions (figure 1). Thus, phases of land use may be correlated to colluvial deposition, which means these deposits can be interpreted as a proxy for human presence, land use and settlement during a specific period of time. The term ‘geomorphodynamic activity’ was coined by Rohdenburg [13,14] to describe phases of increased slope erosion triggered by an accentuated precipitation regime and thereby changed vegetation cover during the Pleistocene. In this study, the terms geomorphodynamic activity or stability are used to describe whether slope deposits are being eroded or stable. Thus, they are broken down to a local or site-specific scale to describe phases of soil erosion and colluvial deposition which are not necessarily connected to climate, but instead are linked to land use changes triggering soil erosion. Owing to slope stability and the supporting influence of vegetation, pedogenic processes take place mainly during stable phases, whereas geomorphodynamic activity leads to redeposition and soil loss [15,16]. The reconstruction of phases with intensified (unsustainable) land use also draws attention to periods of time without colluvial deposition, which separate the different, stratified layers of colluvial deposits. These might have been time periods without agricultural land use or with some kind of low-intensity activities that preserved soil in place. The occurrence of soil erosion and deposition depends on environmental conditions (e.g. topography, soil, climate), population density, technological knowledge and practices (e.g. agriculture, trade, rituals, valuations), and other factors. In addition, anthropogenic colluvial deposits have to be distinguished from natural slope deposits, formed by periglacial processes, bioturbation or soil creep, regardless of land use practices [12,17,18].

This study aims to reconstruct human–environment interactions by analysing land use dynamics in SW Germany through a series of ‘site biographies’ that are primarily based on optical stimulated luminescence (OSL) dating of colluvial deposits and accelerator mass spectrometry (AMS)-^14^C dating of included charcoal fragments. A site biography can be understood as the chronostratigraphy of colluvial deposition and thus is a local reconstruction of deposition and land use phases, including soil and environmental properties, such as organic carbon content, particle size distribution, heavy metal content and topography. Studying such site-specific soil mosaics in space and over time can enhance our understanding of human–environment relationships (i.e. land use dynamics). After a thorough archaeological investigation of settlement history, we used an interdisciplinary approach to select archaeopedological study sites. This allowed us to coordinate the field and laboratory analyses with the interpretation of colluvial soils as records of former land use to provide insights into an expansive archive on the land. The local history of the corresponding slopes is stored in colluvial deposits which can show a high temporal resolution. Previous studies showed that colluvial deposits are most often found in toe- and foot-slope positions but they also reach back slopes or may be stored temporarily in sinks on slopes depending on small-scale topography, precipitation and land use history [7,12,19–22]. The study of many local sites in different landscapes thereby provides an opportunity to reconstruct a regional land use history.

The focus of this study is on soils as a natural resource and basis for land use, especially agricultural land use. The term ‘resource’ is understood as an analytical concept with a constructivist perspective: as a base to create, maintain or alter social relations, units, and identities within the framework of culturally shaped beliefs and practices. It thus includes a wide variety of tangible and intangible means, dynamic social processes of turning something into a resource, and social contexts [23,24]; it thereby includes many possible explanations of pull factors to settle land. The resource concept provides a new analytical approach to interpret archaeological and archaeopedological data about settlement and land use dynamics. It makes it possible to question the natural deterministic model [25] to describe settlement dynamics through time and includes sociocultural explanations. It can be hypothesized that the perception of the quality of a landscape is defined by its usefulness and whether it subsequently was seen and treated as an unfavourable or favourable one.

This paper focuses on the Swabian Jura and its land use history, which will be compared to the Black Forest [26] and the Baar area [19]. The regional focus of the study comprises favourable and unfavourable areas in low mountain ranges for practising agriculture due to the specific conditions of climate, topography and soils. Bourke [27] states that crops and grass species need a minimum mean air temperature of at least 6°C over a period of six months to grow and be harvested. Overall, however, the differentiation between favourable and unfavourable areas [28] has to be understood as a concept to distinguish areas and highlight differences. The attribution of favourable or unfavourable, however, always depends on the perspective and the intention of the user, and it may change with time and context. A favourable landscape for practising agriculture can turn into an unfavourable landscape by soil degradation or climate change. Another scenario could be that agriculture might lose its importance because other economically more valuable raw materials such as iron, silver or gold were found. In the latter scenario the landscape would be seen as favourable for mining and exploitation of raw materials, instead of agriculture, which means a shift of perspective and context. The agriculturally favourable Baar area can also be seen as being rather unfavourable because of a large number of days when temperatures drop below 0°C, dense fog and less fertile soils [29], when compared with other more favourable, loess-covered areas nearby with warmer temperatures and more fertile soils. Unquestionably unfavourable landscapes, however, are the Black Forest and the plateau of the Swabian Jura, currently having low mean annual temperature, high precipitation, infertile soils, and in the case of the Black Forest steep slopes.

The main questions of this paper are:

— How are colluvial deposits distributed on the plateau of the western Swabian Jura and how are they related to past land use?
— How did land use dynamics change through time considering favourable and unfavourable areas in SW Germany?
— What might have been the reason to settle and use certain areas, while others were not used?
— How can geomorphodynamically stable periods be explained, i.e. phases without the formation of colluvial deposits?

### 1.1. Regional setting

**Figure 2.**
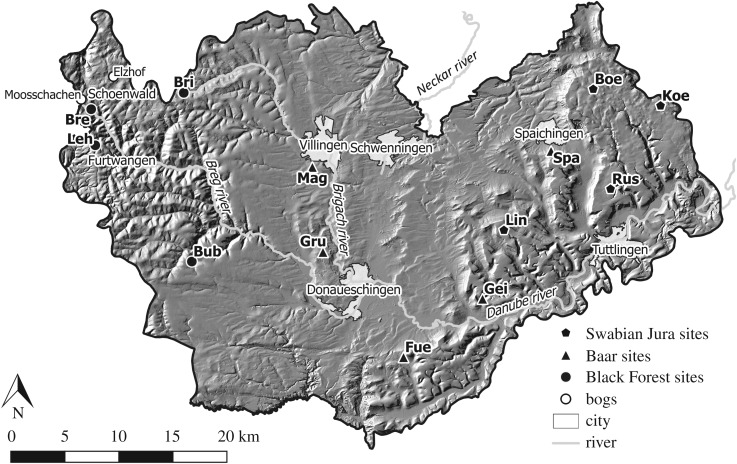
Study area in SW Germany with all archaeopedological and palynological study sites, major rivers and cities. The site names are abbreviations usually from the nearest villages: Boe, Boettingen; Bre, Breg valley; Bri, Brigach; Bub, Bubenbach; Fue, Fuerstenberg; Gei, Geisingen; Gru, Grueningen; Koe, Koenigsheim; Leh, Lehmgrubenhof; Lin, Lindenberg; Mag, Magdalenenberg; Rus, Russberg; Spa, Spaichingen. The Swabian Jura and Black Forest are considered to be unfavourable areas, whereas the Baar is the favourable area. The background map depicts the relief [30].

The study area is located in SW Germany and includes the southeastern Black Forest, the Baar and the western Swabian Jura (figure 2). The focus of this paper lies on four archaeopedological sites situated on the plateau of the Swabian Jura, a low mountain range of Jurassic origin belonging to the cuesta landscape. The high plateau of the Swabian Jura, with an inclination mostly below 10%, has several peaks of about 1000 m altitude and consists of plateaus separated by rivers. The mean annual temperature is about 4–7°C and the mean annual precipitation is 1000 mm. It is characterized by limestone, covered by periglacial slope deposits and colluvial deposits. At present, the environmental conditions are harsh and unfavourable for practising agriculture. The cuesta drops abruptly about 200–400 m in altitude into the Baar area, having rather favourable environmental conditions [19]. The southeastern Black Forest is characterized by a high relief intensity, high annual precipitation, low mean temperatures and acidic soils [26].

The four study sites on the Swabian Jura are presently used as grassland, cropland or woodland and have slightly different site characteristics (table 1). The sites Boettingen (Boe), Koenigsheim (Koe) and Russberg (Rus) belong to an area called ‘Grosser Heuberg’ on the Swabian Jura, which has a long settlement history, despite rather unfavourable conditions. The site Lindenberg (Lin) is located further west.

**Table 1.** Location and characteristics of Swabian Jura sites. The compilation originates from fieldwork and information about geology [31] and soils [32].

site	Russberg (Rus)	Lindenberg (Lin)	Koenigsheim (Koe)	Boettingen (Boe)
UTM	32U485944	32T475990	32U490366	32U484337
	5319526	5315709	5327191	5328676
geology	dense late Jurassic limestone (Unterer Massenkalk); marl; Holocene slope deposits	layered late Jurassic limestone (Wohlgeschichtete Kalke Formation); Holocene slope deposits	late Jurassic dolomite and crystalline limestone (Zuckerkornkalk); dense limestone (Unterer Massenkalk); quaternary slope and weathered deposits	layered late Jurassic limestone (Wohlgeschichtete Kalke Formation); Quaternary slope and weathered deposits
main soil types (WRB)	Regosol; Cambisol	Regosol; Cambisol	Cambisol; Regosol	Cambisol; Regosol
vegetation	woodland; cropland	woodland; cropland	grassland; cropland	grassland
topography	W-facing; inclination 2–20%; beginning of a V-shaped valley	upper end of depression on plateau; SE-facing; 2–10% inclination	S-facing; inclination 2–5%; back- to foot-slope position of a depression	S-facing; inclination 2–4%; back-to foot-slope position
hydrology	no surface water; no drainage	no surface water; no drainage	no surface water; no drainage	no surface water; no drainage
altitude	830–850 m.a.s.l.	900–920 m.a.s.l.	870–890 m.a.s.l.	930–940 m.a.s.l.
land use	forestry; farming	forestry; farming	farming; hay meadow	hay meadow

### 1.2. Settlement and land use history of the western Swabian Jura and the Heuberg

There are no known Mesolithic (9600–5500 BCE) or Early Neolithic (5500–5000 BCE) sites on the high plateau of the Swabian Jura in the study area. The oldest find is a so-called ‘shoe-last celt’ (Schuhleistenkeil) dated to the Early Neolithic, found in the vicinity of Talheim, near the Lindenberg site. This find is interpreted as a sign of temporary land use in the valleys of the western Swabian Jura during the Early Neolithic [33]. Further single finds, e.g. a stone axe, were on the plateau of the Swabian Jura and were dated to the Neolithic [33,34]. The earliest archaeological evidence of a settlement on the western Swabian Jura dates to the Final Neolithic (2800–2150 BCE) and consists of pottery fragments, stone axes and flint artefacts found on the Dreifaltigkeitsberg near Spaichingen [35,36].

There are no archaeological Early Bronze Age (2150–1550 BCE) sites known on the Swabian Jura. Temporary land use is indicated by a burial site found in Boettingen dated to the Middle Bronze Age (1550–1300 BCE) [37]. There are a few Late Bronze Age (1300–1200 BCE) sites known in river valleys, but none on the Swabian Jura itself. However, during the late Urnfield period (Urnfield period 1200–800 BCE), archaeological finds point to an expansion of populated areas, indicated by several hilltop settlements where intensified land use was practised on the western edge of the Swabian Jura, coupled to land use in adjacent narrow river valleys. The expansion of the populated area from the favourable Baar area to unfavourable areas such as the Swabian Jura has been explained by external pressures, for example, drastic climatic changes or overpopulation, and human conflicts in the Baar [37–39]. This explanation, however, is not supported by local archaeological data [40].

Hoards and the deposition of metal artefacts in bogs as well as at springs of larger rivers such as the Neckar reveal a fundamental change in the perception of landscapes during the Urnfield period. This development can also be seen on the high plateau of the Heuberg. Here, two sites, Götzenaltar and the Heidentor near Koenigsheim and Boettingen, may have been used for ritual purposes during the Urnfield period. The Götzenaltar is a large limestone block, located on a small hill. The Heidentor is a natural rock formation with the shape of a wide gate. This site is located on a steep slope at the edge of a mountain range called ‘Oberburg’ above the village of Egesheim [35]. Numerous pottery fragments suggest that repeated visits were made to both sites [37,41]. At the Heidentor the pottery was thrown through the rock formation downslope. The rock formations can be considered ritual sites [40], because they fulfil the criteria of extraordinariness and repetition introduced by Colpe in 1970 [42].

With the transition to the Iron Age (800–±10 BCE/CE), the land use pattern changes considerably on the Heuberg. A reduction of settlement activities can be observed on the Swabian Jura, because only one site on the Heuberg actually dates to the early Hallstatt period (Hallstatt period 800–450 BCE), but during the late Hallstatt period, settlement activity increases again [37]. A change in the conceptualization of landscapes can be interpreted from an increasing number of ritual sites on the western Baar and Swabian Jura [43–45]. Examples of ritual sites are the Götzenaltar and the Heidentor, used during the Iron Age as well as during the Urnfield period [33,46], or a large deposit of sherds from pottery vessels near Lindenberg [47,48]. The distribution of settlements and burial sites on the Heuberg indicates a land use pattern related to the ritual use of the Heidentor. The majority of the settlements are located in the southwestern part of the Heuberg, while the burial mounds lie mainly in the area between the settlements and the Heidentor and no sites could be found in the direct vicinity of the Heidentor. Thus, the landscape can be differentiated in the ‘landscape of the living’ (settlements), the ‘landscape of the dead’ (burial sites) and the ‘void’ (no sites) [40]. This pattern of land use was culturally constructed with the intention to keep the ritual site in spatial and cultural seclusion, which is typical for places used for transitional rituals. The Latène period (450–±10 BCE/CE) is characterized by a decline of settlement activities on the Swabian Jura, during which very few settlements, such as the one on the Dreifaltigkeitsberg near Spaichingen, are still populated [36]. On the Heuberg, the seclusion of the Heidentor was maintained until the end of the middle Latène period. As soon as the ritual activities stopped, the settlement pattern changed, and the formerly empty areas close to the Heidentor were populated during the late Latène period [40].

During the Roman Empire (±10 BCE/CE–375 CE) settlements were concentrated on the Baar and the adjacent river valleys. Other than a Roman coin hoard found in the eighteenth century, there is hardly any evidence for Roman settlements on the Swabian Jura [49]. With the Merovingian period (450–750 CE) comes an intensification of land use in the large valleys of the Danube, Breg and Brigach rivers, and smaller rivers on the Swabian Jura, in contrast to the sparse archaeological sources on the Heuberg. During the Middle Ages (450–1500 CE) the settlement pattern changed and was dominated by scattered settlements without obvious concentration in certain landscapes. During the High Middle Ages (750–1250 CE) settlement activities intensified on the Heuberg.

## 2. Material and methods

### 2.1. Field

Fieldwork was carried out from 2013 to 2015 with the permission of landowners and tenants. Fieldwork included the description of 15 soil profiles on the Swabian Jura and additionally 53 soil pits in the Baar and the Black Forest. The soil profiles were described following the German soil classification system [50], the FAO 2006 [51] and the WRB 2015 [52]. The German classification system uses the horizon designation *M* (M = Lat*. Migrare*, to migrate) for anthropogenic colluvial horizons lacking other pedogenic properties. As it is important to differentiate colluvial horizons from others with different pedogenic development, we use the M horizon together with the FAO nomenclature. German soil types were translated into WRB using translation software [53,54] and a manual check.

The soil profiles are located along catenas reaching from the upper slope to foot-slope positions. Catenas represent a series of soil profiles along a slope having different characteristics due to differences in topography, parent material, drainage, erosion or deposition [55]. The locations of catenas and soil profiles were chosen to represent a stratigraphy of colluvial deposits in close proximity to known prehistoric activities. Samples for dating were collected from colluvial deposits showing the most detailed pedostratigraphy and being characteristic for the site. To prevent sampling bias for specific time periods, soil samples for dating were collected consistently from all soil horizons, in which sampling was possible.

At the Swabian Jura sites a total of 166 bulk samples and 128 volumetric samples (each consisting of 3 × 100 cm^3^ subsamples) were taken from all horizons. From each colluvial horizon, the upper 5 cm were sampled separately, and colluvial horizons thicker than 20 cm were split into thinner sampling units. The sampling and dating strategy allows us to reconstruct the chronology of colluvial deposition and to reconstruct phases of land use at different sites.

### 2.2. Laboratory

Total C and N contents (mass %) were analysed using oxidative heat combustion at 1150°C in a He atmosphere (element analyser ‘vario EL III’, Elementar Analysesysteme GmbH, Germany, in CNS mode). Soil organic C content (SOC) was determined using: SOC = C_total_ − CaCO_3_ × 0.1200428. Bulk density (g cm^−3^) was gravimetrically determined (cf. [56]). Carbonate content was determined volumetrically by CO_2_ evolution using a calcimeter (‘Calcimeter’, Eijkelkamp, Giesbeek).

To estimate depositional ages of the colluvial sediments, OSL dating was applied, using opaque steel cylinders with a diameter of 4.5 cm for sampling. For equivalent dose (D_e_) determinations, the coarse grain (90–200 µm) quartz fraction was prepared and measured with a single-aliquot regenerative-dose (SAR) protocol after Murray & Wintle [57]. All luminescence measurements were carried out at the luminescence laboratory of the Justus-Liebig-University in Giessen, using a Freiberg Instruments Lexsyg reader [58]. For data analysis, the R luminescence package [59] was used.

To avoid modern bleaching by bioturbation, soil material from the upper 30 cm of the profiles was not sampled for OSL dating. In consequence, colluvial deposition of the Modern era might be underrepresented. This might also apply to older colluvial deposits, because of the generally better preservation of younger deposits. However, the general suitability of OSL dating on colluvial deposits is shown in numerous studies, despite issues of partial bleaching (e.g. [6,60,61]). Most soil samples have good properties for luminescence dating, showing a bright luminescence signal. Therefore, small aliquots with a diameter of 1–2 mm were measured. In the case of significant skewness of the equivalent dose distribution, a minimum age model [62] was used. Skewness can result from partial bleaching, e.g. by bioturbation.

AMS-^14^C dating of charcoal fragments found within the colluvial deposits was carried out at the laboratories of Erlangen, Jena, Mannheim and Poznan. The pretreatment was done using the ABA (acid–base–acid) or, in case of samples measured in Jena, by an ABOx (acid–base-oxidation) procedure [63]. The conversion of the ^14^C isotope ratios in calendar and calibrated ages was done with OxCal 4.2 using the IntCal13 calibration curve [64,65]. If the pretreatment omitted all contaminations and the charcoal fragment was incorporated when the colluvial deposit formed, the age of the charcoal represents the age of the layer plus the time span from the death of the tree to deposition, i.e. the charcoal age is an upper limit for the age of the colluvial horizon.

The basic assumption for the interpretation of charcoal ages is that no relocation within the soil profile occurred. Occasionally, we encountered sample ages which appeared to be out of sequence in relationship to other dated samples within a soil profile. In those cases, where the majority of ages formed a clear stratigraphic sequence, and certain charcoal samples dated to much older or younger times than expected due to their sampling location within the sequence, we assumed relocation of those samples by natural processes of bioturbation or redeposition. Age inconsistencies may also be due to the use or reuse of old timber because the samples date to the time when the tree grew, rather than the time when the wood was processed and used. These confounding effects can also explain charcoal ages which are older than OSL ages.

Because of these complications, the presence of charcoal in a colluvial stratum may indicate human burning activity rather than the age of colluvial deposition. However, it is important to consider the possible deposition conditions by assessing the abundance and distribution of charcoal within the soil profile before inferring either a natural or anthropogenic cause. It is assumed that many isolated charcoal fragments appearing in a soil stratum are more probably a result of consecutive inputs and anthropogenic origin. By contrast, layered charcoal fragments more probably indicate a natural deposition following e.g. a forest fire.

The radiocarbon calibration process can also introduce additional errors if particular ages are associated with problematic parts of the calibration curve (‘wiggles or nonlinearities of the calibration curve), which result in extremely large and non-normal standard error estimates, even for very precisely dated samples. Another factor limiting the explanatory power for older periods might be that younger charcoal samples can be overrepresented because of better preservation and an increasing probability of destructive processes such as erosion and weathering [66–68].

Thus, radiocarbon ages from charcoal fragments could be older, younger or of the same age as OSL samples, depending on site taphonomy and age calibration. The true age of the formation of colluvial deposits is not necessarily dated with the radiocarbon or luminescence method. Because of the difficulties in interpreting the chronologies on individual soil profiles, we employed a statistical approach for analysing radiocarbon and OSL ages that involved calculating summed probability density (SPD) plots [69,70].

Only the available radiocarbon and OSL ages from colluvial layers with a high reliability, based on the comparison of other luminescence and ^14^C ages, and the stratigraphic context, were used in this process. To meet the critique on SPDs [71], sampling should be representative in a way that the probability of having a sample dating to a certain period should have the same relation to the number of sites for all periods [72]. Soil pit locations were purposefully sampled from archaeological contexts; however, within each soil pit, soil material for dating was sampled from the top to the bottom of the vertical pit wall, and from within each identifiable and dateable layer. Because of this, the distribution of the age samples represents a continuous, temporal sample and, therefore, the resulting SPD curves from the ^14^C and OSL ages and error distributions are valid and representative profiles of the colluviation intensity of these sites through time. To calculate SPDs, uncalibrated radiocarbon ages and errors were used and calibrated using the statistical software package Bchron [73] and the calibration curve IntCal13 [65]. The SPD for the OSL ages was generated by sampling from a Gaussian distribution for each date where the OSL ages and their standard errors define the mean and standard deviation of each distribution. The different age probability curves are summed and plotted.

To contextualize our study area in the larger region, we extracted 737 Neolithic and Early Bronze Age radiocarbon ages from the RADON database [74] and calculated a regional SPD. To get a representative dataset, we included ages from following areas: southwest Germany (Baden-Württemberg *n* = 556), the Swiss lowlands (Schaffhausen *n* = 18, Zürich *n* = 100, Basel-Stadt *n* = 0, Basel-Land *n* = 14, Thurgau *n* = 14, Aargau *n* = 8, Solothurn *n* = 3, Jura *n* = 10) and eastern France (Elsass *n* = 0, Franche Comte *n* = 13, Lothringen *n* = 0, Haut Rhin *n* = 0).

## 3. Results

The results of the archaeopedological project are archived online at Dryad [75]. This repository contains the overview from the project ‘Favour–Disfavour? Development of Resources in Marginal Areas' as part of the collaborative research centre SFB1070 ResourceCultures. The results [75] contain the field description of all 68 soil profiles, the laboratory results of 728 bulk soil samples, and a total of 47 OSL and 93 radiocarbon ages. The focus of this paper is on four sites with a total of 15 soil profiles located on the Swabian Jura.

### 3.1. Colluvial deposits on the Swabian Jura

The field analyses of colluvial deposits usually lead to a site biographical interpretation of soil development, colluvial deposition, and a wider interpretation about environmental change and land use. This process to assess the geomorphodynamic situation leads to the interpretation of geomorphodynamically stable and instable phases, concerning soil erosion and deposition triggered by land use. Following our interpretation, geomorphodynamically stable phases are characterized by thin or no colluvial deposits, whereas thick and multilayered colluvial deposits containing many artefacts indicate geomorphodynamic instability, most probably caused by land use (figure 1). Multilayered colluvial deposits can be covered by deposits having a fully developed topsoil horizon indicating a phase of environmental stability and thus surface horizon development and pedogenic processes. If this surface horizon is covered by new colluvial deposits, it can be concluded that the site has undergone alternating phases of geomorphodynamic instability and stability, in other words alternating phases of land use including colluvial deposition and phases of no land use with predominantly natural vegetation cover or phases where sustainable land use techniques were used. This site biography can be interpreted using ages, archaeological knowledge, soil chemical and physical results, and further (palaeo)environmental data to reconstruct site-specific land use dynamics.

#### 3.1.1. Lindenberg

**Figure 3.**
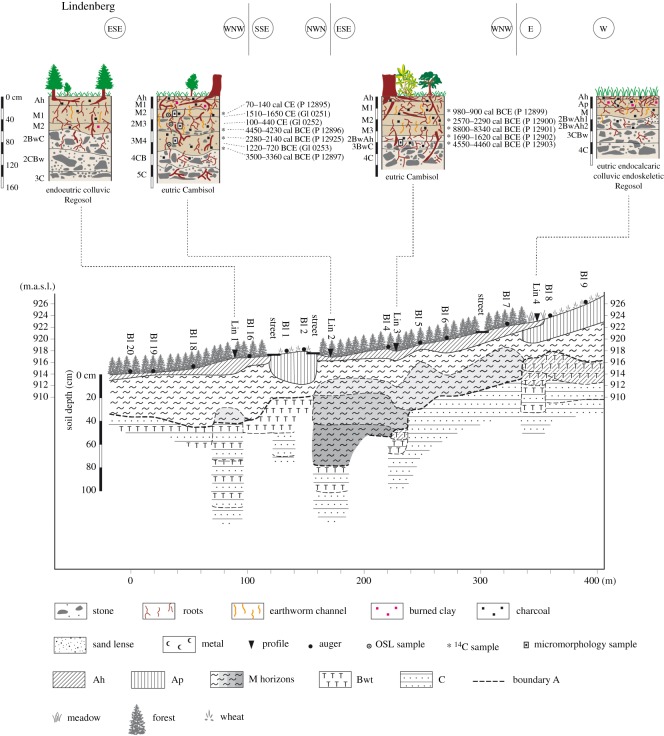
Catena and soils at the Lindenberg site, situated on the back slope.

The soils at the Lindenberg site (figure 3) are developed in sediments on limestone covered by colluvial deposits. The two upper soil profiles (Lin3, Lin4) consist of colluvial material covering a clay-rich (greater than 60% clay) soil type, Terra fusca. The subsoil is dominated by a very high limestone content. The lower profiles Lin1 and Lin2 do not include Terra fusca material. Lin2 has the thickest colluvial deposits with four layers. Lin1 is partly eroded, which can be explained by its position on the shoulder of a slope of a V-shaped valley of the Weissenbach, a Danube tributary. The archaeological finds in the direct vicinity date to the Neolithic and Bronze Age, and then to the Roman Empire, the Middle Ages and the Modern times. The pattern of archaeopedological ages is almost the same. The earliest human presence manifests itself through dated charcoals during the Neolithic, but more intensive land use leading to colluvial deposition sets in only during the Bronze Age. At the Lindenberg site no colluvial deposits or charcoals dating to the Middle Ages were found. The Early Mesolithic charcoal (cal BCE 8800–8340, P12901, Lin3) points to very early land use or natural forest fire.

#### 3.1.2. Boettingen

**Figure 4.**
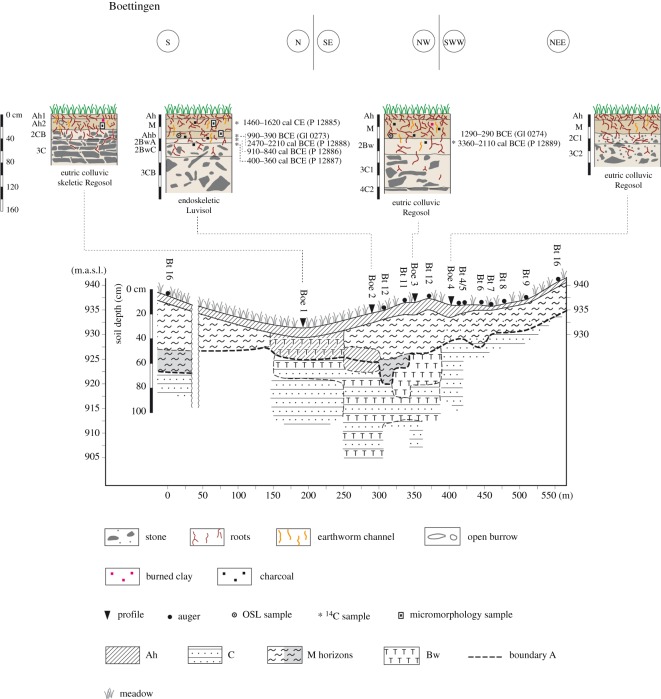
Catena and soils at Boettingen, situated from the toe slope to the back slope.

The area around Boettingen (figure 4) is known to have been used since the Bronze Age. The thickest colluvial deposit, however, includes only two colluvial layers (auger Bt11, Bt12) at the foot of a slightly terraced slope. The profile (Boe2) in the same position shows a former surface horizon with high SOC content underneath the colluvial deposit and above the subsoil. The colluvial deposit dates to the Bronze/Iron Age (BCE 1290–290, GI0274, Boe3) and contains Modern and Neolithic charcoals (cal CE 1460–1620, P12885, Boe2; cal BCE 2470–2210, P12888, Boe2). The flat areas (Boe1) show bioturbation by animals and smaller soil organisms down to 30 cm when a mixed horizon of subsoil and weathered limestone rocks begins.

#### 3.1.3. Koenigsheim

**Figure 5.**
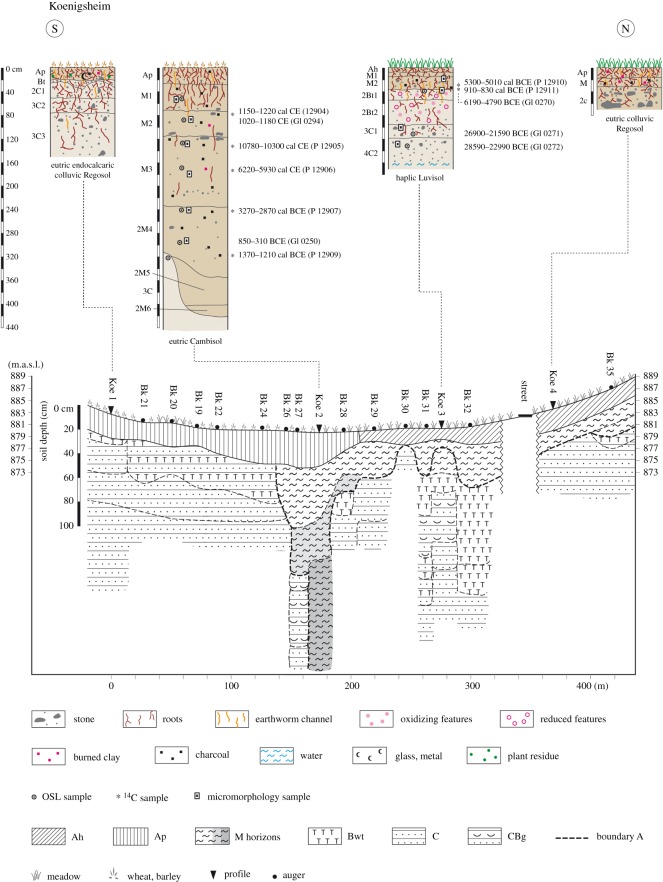
Catena and soils at Koenigsheim, situated in a depression.

The Koenigsheim site (figure 5) is situated in a shallow depression, surrounded by mixed and coniferous forest and the village of Koenigsheim. Light brown colluvial deposits are mostly limited to a depth of 40 cm (Koe4, Koe3) or to the plough depth (Koe1). Underneath the colluvial deposit subsoil horizons occur, which were formed in periglacial layers. Lower horizons especially are dominated by coarse limestone fragments. A special feature of the catena is the soil profile Koe2, a completely filled doline. The doline is around 3 m deep and now filled with dark brown clayey colluvial deposits, which can be separated into four layers. The dark brown soil material does not occur in the surrounding area. The OSL ages suggest that the subsidence of the doline might have taken place in the transition of the Bronze to Iron Age. The lower part of the lowest M horizon (M4, 233–320 cm) dates to the Hallstatt period (BCE 850–310, GI0250) and contains a Middle to Late Bronze Age charcoal (cal BCE 1370–1210, P12909). The upper part of the same horizon contains a Late Neolithic charcoal (cal BCE 3270–2870, P12907) and the overlying M3 horizon even contains Late Mesolithic and Late Palaeolithic charcoals (cal BCE 6220–5930, P12906; cal BCE 10780–10300, P 12905). It can be assumed that the M3 horizon reflects a balancing of the relief and functioned as a soil surface during the Middle Ages. The doline must have been filled with pedosediments (redeposited soil) within around 1000 years from the Iron Age to the Roman Empire, since the OSL and calibrated ^14^C ages of the M2 horizon date to the High Middle Ages (CE 1020–1180, GI0249; cal CE1150–1220, P12904).

Archaeological finds point to land use during the Urnfield period, the Iron Age and from the Middle Ages onwards. This pattern is supported by the archaeopedological ages, but additionally charcoals date back to the Neolithic and even Mesolithic.

#### 3.1.4. Russberg

**Figure 6.**
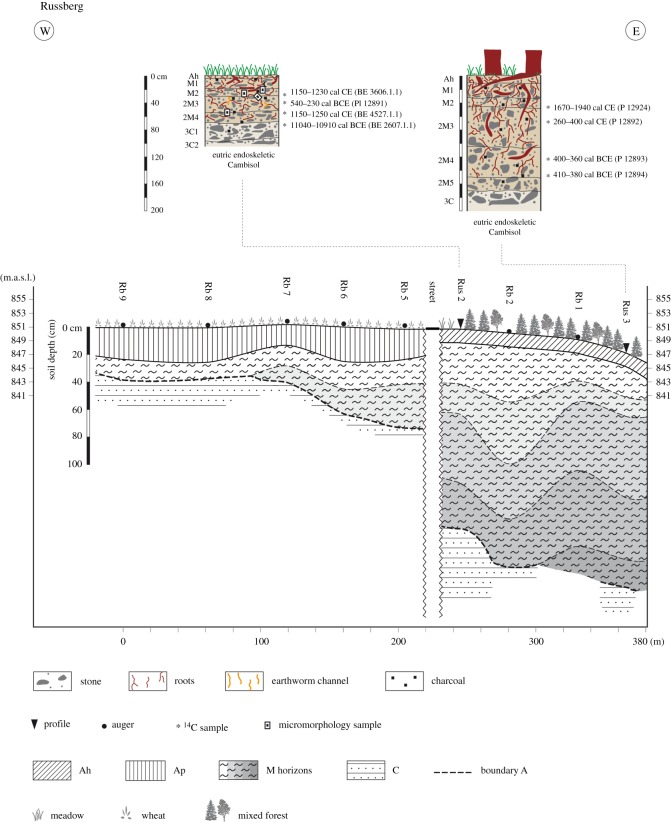
Catena and soils near Russberg, situated on the shoulder and the back slope.

The soils at the Russberg site (figure 6) are developed from the same geology as in Koenigsheim and the settlement history is also very similar, indicating land use during the Hallstatt period and from the Middle Ages onwards. The soil profiles are situated on a plateau, which is covered by shallow colluvial deposits overlying limestone-rich subsoils. Only the upper end of a v-shaped valley of a small Danube tributary contains thick multilayered colluvial deposits (Rus2, Rus3). And the rest of the v-shaped valley was exposed to intense erosion and has only thin soils. Charcoal ages from these sites support archaeological findings and date to the Iron Age (cal BCE 400–360 P12893, Rus3; cal BCE 410–380, P12894, Rus3; cal BCE 540–230, P12891, Rus2), Roman Empire (cal CE 260–400, P12892, Rus3), Middle Ages (cal CE1150–1230, BE3606.1.1, Rus2; cal CE 1150–1250, BE4527.1.1, Rus2) and Modern times (cal CE 1670–1940, P12924, Rus3). The lowest colluvial horizon of Rus2 contains an Early Mesolithic charcoal (cal BCE 11040–10910, BE3607.1.1), which is most probably relocated within the soil profile, e.g. by bioturbation.

### 3.2. Chronostratigraphy

**Figure 7.**
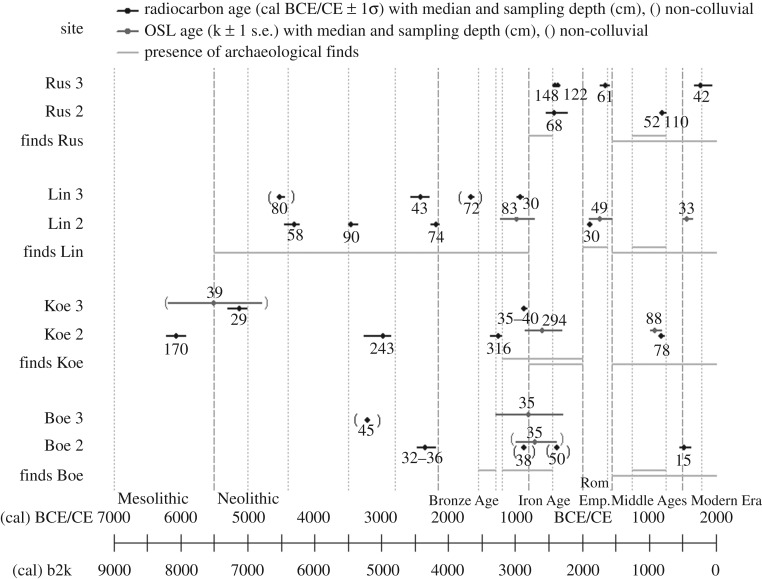
AMS-^14^C and OSL ages of colluvial deposits on the western Swabian Jura compared to known archaeological finds at each site from the Neolithic to the Modern Era. The dashed lines mark archaeological epochs and the dotted line archaeological periods.

The chronostratigraphy of colluvial deposits, inferred from dated charcoals, on the Swabian Jura indicates that intensified land use and land use change began in the Mesolithic at the Koenigsheim, Russberg and Lindenberg sites. The onset of continuous land use (cultivation) seems to have started during the Neolithic (figure 7). Neolithic charcoals were found in colluvial deposits at Lindenberg, Koenigsheim and Boettingen. The oldest OSL ages of colluvial deposits, however, date to the Late Bronze Age and Urnfield period. This onset of luminescence ages indicates an intensification of agricultural land use or land use change from the Late Bronze Age to the Hallstatt period. Land use is not detectable during the Roman Empire at the sites Boettingen and Koenigsheim using colluvial deposits as a proxy, which indicates the absence or low impact of human activities on the environment. Charcoals dating to the Iron Age were found in colluvial deposits at the Russberg site. At Lindenberg and Russberg, colluvial deposits and charcoals were found dating to the Roman Empire. Despite the well-documented archaeological knowledge of the Middle Ages, medieval colluvial deposits were only found at Russberg and Koenigsheim.

Even though the environmental conditions on the western Swabian Jura are very similar, differences in land use dynamics between the investigated sites can be inferred from colluvial deposits and dated charcoals.

**Figure 8.**
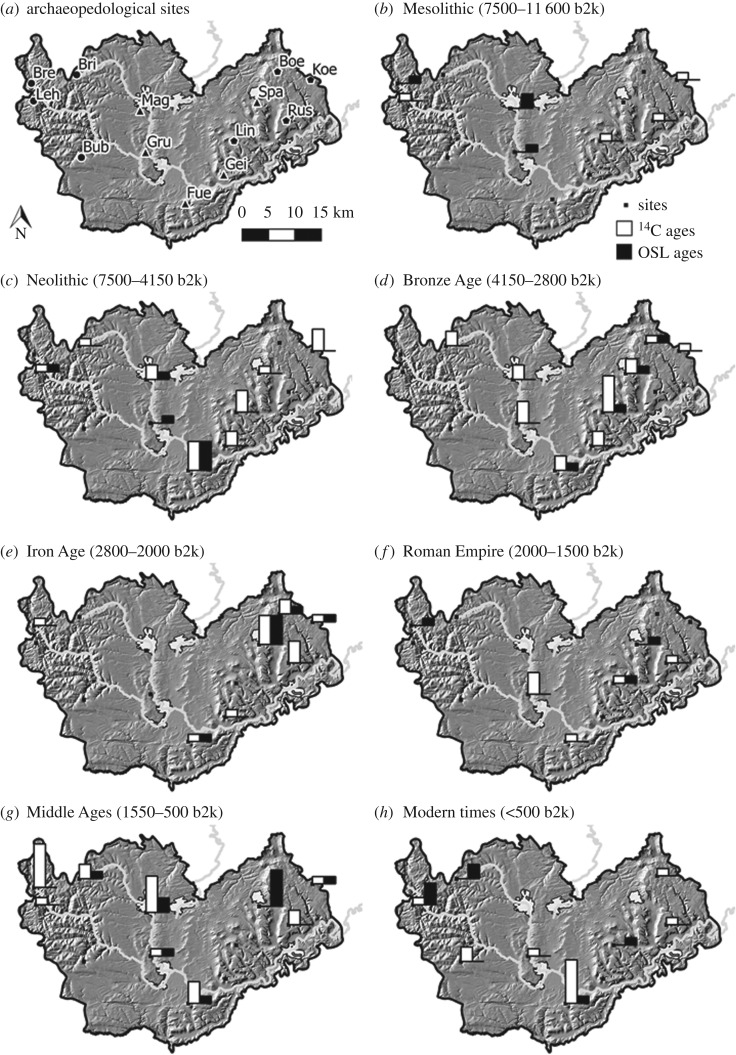
(*a*–*h*) AMS-^14^C and OSL ages across the study area in different periods from the Mesolithic to Modern times. The height of the black bars indicates the number of OSL ages at the respective site. The range is from 1 to 5. The height of the white bars indicates the number of ^14^C ages at the site. The range is 1 to 6. The Swabian Jura sites (pentagon) and the Black Forest sites (circle) are situated in unfavourable areas, whereas the Baar sites (triangle) are characterized as favourable (in *a*). The background map depicts the topography after [30].

The comparison of ages of colluvial deposits from the Swabian Jura with ages of colluvial deposits from the Black Forest (figure 8) shows that these two unfavourable areas were differently settled and used. The southeastern Black Forest shows only local land use before the Middle Ages, whereas the Swabian Jura seems to have had similar land use dynamics as the western Baar. Human land use activities were detected to have been early at the central Baar area, but no colluvial deposits were found during the Iron Age.

**Figure 9.**
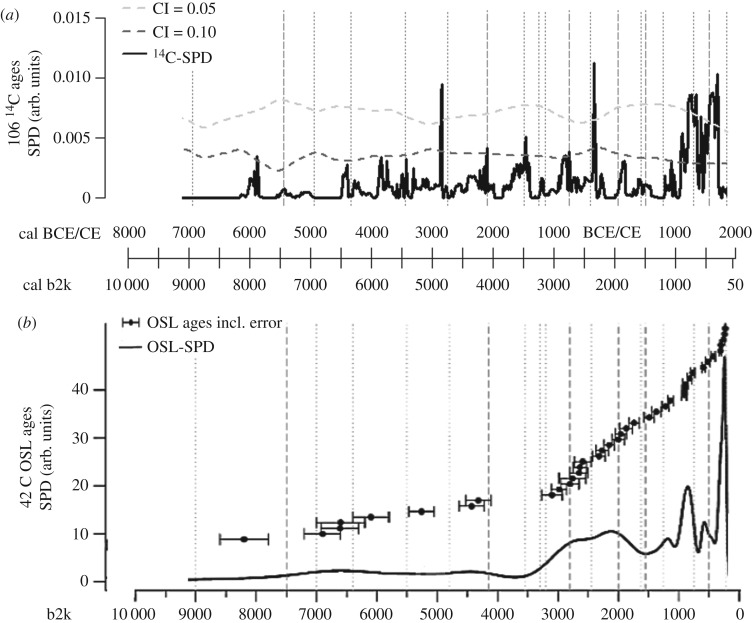
SPDs calculated for the whole study area including the Baar, Black Forest and Swabian Jura. Periods are indicated by dotted lines. (*a*) SPD using 106 ^14^C ages, (*b*) SPD using 42 OSL ages.

Calculating SPDs using all ages from colluvial deposits found in the Baar, the Swabian Jura and the Black Forest results in a ^14^C-SPD (figure 9*a*) showing many peaks within the confidence intervals. These may result from the calibration error. It is shown that the probability of charcoal occurrence increases during the Neolithic, indicating increasing human activity in the study area, analogue to the interpretation of charcoal as an indirect proxy for demographic levels [69]. A cluster of peaks above the confidence intervals (0.05 and 0.10), e.g. during the Bronze and Iron Age and from the High Middle Ages to the Early Modern period, reliably indicate phases of high human activity.

Based on the OSL-SPD (figure 9*b*), the probability of colluvial deposits is very low during the Neolithic, but shows an increase from the Late Bronze to the Iron Age. The possibility of colluvial deposition is even higher during the High Middle Ages and the Early Modern period.

**Figure 10.**
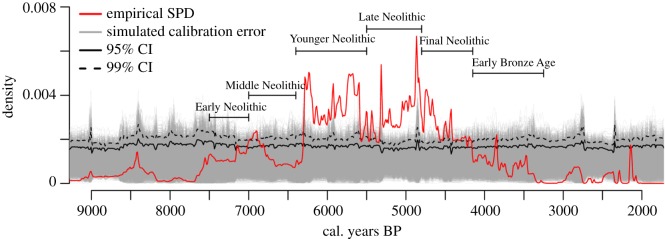
SPD of radiocarbon ages from the RADON database [74], showing the confidence intervals and periods. The RADON database focuses on ages from the Neolithic and Early Bronze Age.

To put the data into a larger context, an SPD of radiocarbon ages from southwestern Germany, eastern France and the Swiss lowlands (from the RADON database [74]) was calculated (figure 10). The database and thus the SPD is focused on the Neolithic and Early Bronze Age and shows a clear increase of human impact at the transition from the Early to the Middle Neolithic (around 5000 cal BCE), followed by a rapid decline during the Middle Neolithic. A strong signal dates to the Younger and Late Neolithic, which can be separated into two phases because the SPD drops below the confidence interval and into the error margin. The Final Neolithic and Early Bronze Age show a general decline in radiocarbon ages and thus human activity; however, it should be noted that the RADON database focuses on the Neolithic, and the decline apparent in this figure may be in part due to edge effects.

## 4. Discussion

Regarding the pre- and early historic land use, radiocarbon dating of charcoals in colluvial deposits and OSL dating of colluvial deposits provide numerous new insights, because they add knowledge about time periods so far missing in the archaeological record. Our data show the value of using two different dating methods. Radiocarbon dating of charcoals is precise but difficult to interpret in relation to the timing of the formation of colluvial deposits because the charcoal fragments are prone to dislocation (the extent of which cannot be measured) by bioturbation within the soil, superficial relocation with run-off water or later incorporation into the soil. In most cases the dating of charcoals gives a maximum age of the formation of the related colluvial layer, which in some cases can be more than 3000 years older than the corresponding OSL age of the colluvial layer (e.g. [6,76,77]). In some cases, the results of both dating methods yield similar or even the same results, which gives a robust chronostratigraphy of colluvial deposits (cf. profile Mag1 in [19]). Both dating techniques give physically correct ages; however, given the dynamics of colluvial deposition processes, OSL ages seem to be—in most instances—more reliable in giving a model age of the period of time during which the dated colluvial layer was formed. However, there might be a time lag between the trigger of soil erosion and the time of deposition. In any case, all dating results have to be discussed and evaluated in their geomorphological/pedological context. The compilation and comparison with other data from the same region will give a robust regional chronostratigraphy of colluvial deposits. An additional factor is the time lag between the age of the settlement, or respectively, the time period during which the slope was used and the time when the colluvial layer was deposited. However, the link between colluvial deposition and land use or settlement activities is not always reliable. This study showed the concurrence of colluvial deposits and known archaeological sites in the area, but we could not establish a direct relation to a certain settlement, mainly because there are only few studied ancient settlements in the area. More studies are needed to establish this relation between archaeologically known settlements and land use sites and colluvial deposition in the direct vicinity.

### 4.1. Land use dynamics on the Swabian Jura, the Baar and the Black Forest

#### 4.1.1. Mesolithic

The Mesolithic overview (figure 8) shows that there are two sites on the Baar and one in the Black Forest having Mesolithic OSL ages. The OSL age from the Black Forest is likely to result from a contamination of the colluvial sample with underlying periglacial sediment. The Mesolithic samples of the Baar might be interpreted as the beginning of colluvial deposition and thus long-term and intensive land use during the Mesolithic in the area. Traditionally, Mesolithic communities are not expected to practice agriculture or clear forests, but they disturbed the natural forest vegetation, which is visible in many pollen diagrams through the presence of microcharcoals. These microcharcoals are linked to burning and forest clearance [78–80]. Divišová & Šída [81] offer the explanation of forest clearings as social phenomena out of fear and anxiety about the environmental surroundings. The increased use of wild plants during the Mesolithic [81,82] might have led to local agriculture and the transition to the Neolithic culture [69,83,84]. There is a long-time discussion about the interpretation of proxies for Mesolithic agriculture: the practice of agriculture during the Mesolithic is inferred from cereal pollen found in bogs in central Europe, especially in Switzerland [85,86]. Behre [87] contrasted that the interpretation of Mesolithic agriculture is often based on single pollen finds described as cereal pollen, where it could also be pollen of wild forms of cereal-type pollen and is, therefore, not reliable as a proxy for agriculture.

Charcoals from Russberg, Koenigsheim and Lindenberg indicate land use during the Mesolithic on the Swabian Jura. The lack of archaeological Mesolithic sites on the Swabian Jura is most probably a result of the nomadic way of life during this epoch [88]. Forager societies usually had smaller settlements and thus less influence on the environment, therefore it is much more difficult to detect their former presence in an area [69]. In this way, Mesolithic charcoals can only be an indirect indication of human presence or they can be interpreted as being caused by natural processes such as wildfires.

Our archaeopedological results add another proxy to the discussion about Mesolithic agriculture. As we interpret the finding of many charcoals in soils as an indirect proxy for human presence and land use, some forms of land use can undoubtedly be shown for the Mesolithic. The Mesolithic OSL ages can be interpreted as a reference of open landscapes, probably due to agricultural land use, and thus as an early development or adaptation to a new lifestyle (cf. [83]). The beginning of colluvial deposition and the increased number of charcoals with the transition from the Mesolithic to the Neolithic clearly points at intensive and continuous land use and consequently at sedentary and agricultural lifestyles.

#### 4.1.2. Neolithic

The onset of an area-wide sedentary and agricultural society can be dated to the Neolithic. The number of Neolithic charcoals on the Swabian Jura and the Baar is higher than in the western study area (figure 8). Neolithic charcoal fragments of Lindenberg, Koenigsheim and Boettingen can be interpreted as proxies of temporary land use on the Swabian Jura, possibly within the framework of a seasonal pasture economy. So far, land use was only known from the Heuberg area, which is the first known indication of land use at these sites during the Neolithic. Pollen records from the southeastern Black Forest [26] date the first occurrence of human indicator pollen to the Younger Neolithic. The oldest phase of colluvial deposition on the Baar dates to the Younger Neolithic (*ca* 3700 BCE, [19]) and correlates with a wetter and colder period [89]. Additionally, decreasing atmospheric ^14^C production rates [90] and increased ice-rafted debris in the Northern Hemisphere [91] indicate these conditions. Temperature reconstructions using lake levels [92] and pollen data [93], in contrast, indicate drier and warmer conditions. Despite these contrasting climate reconstructions, colluviation seems to be triggered by the onset of agricultural land use. The increased signal of human activities during the Neolithic (charcoals, pollen, colluvial deposits and archaeological finds) found across our study area point at an increased regional human impact on the landscape from the Neolithic onwards, which is in agreement with the SPD calculated from the RADON database [74] including samples from a wider region (figure 10).

Shennan, Downey and colleagues [1] date the earliest farming in southern Germany to around 5450 cal BCE and reconstruct the first significant agriculture-driven boom of population density from 5200 to 4950 cal BCE followed by a rapid decline, the bust phase during the Middle Neolithic. The Younger and Late Neolithic are characterized by several minor boom–bust phases and led to a steady decline during the Final Neolithic and Early Bronze Age, which is mirrored by the trend of the RADON-based SPD. The first increase of population levels during the Early Neolithic is shown by Shennan & Edinborough [72] for all of Germany. The difference in our results is the trend during the Younger Neolithic, where only low population levels were reconstructed. Compared to this study, the archaeopedological dates presented in this study seem to have a delayed trend, picturing the peaks in the Late Neolithic. This difference probably results from the smaller dataset of the study area, excluding some favourable areas and well-studied archaeological locations, thus maybe truly depicting later and less intense development in the area. Pollen records from the southeastern Black Forest [26], in contrast, mirror the increase of population with the onset of human indicator pollen.

#### 4.1.3. Bronze Age

OSL ages provide the earliest evidence for land use on the southwestern Swabian Jura during the Early Bronze Age. Archaeological finds point at land use only during the Middle Bronze Age and Urnfield period on the Heuberg, but the archaeopedological OSL and ^14^C ages of Boe3 and Koe2 indicate land use during the Late Bronze Age. For the Urnfield period the archaeological record and archaeopedological data correlate well, indicating land use. Further, intensive land use is indicated on the Baar marking a main colluviation phase (*ca* 1400 BCE, [19]) and also at one site in the southeastern Black Forest (figure 8). The increased colluvial deposition coincides with a cold and humid climate [89] with especially cold summers as reconstructed by pollen data [93], but again low lake levels [92] and an indifferent, global trend of atmospheric ^14^C production [90] and the occurrence of ice-rafted debris [91]. It is the transition to a dry period [94,95].

#### 4.1.4. Iron Age

Archaeological records and archaeopedological data correlate well during the Hallstatt and the Latène period on the western Swabian Jura. There is first evidence of land use at the Russberg site. The mining of bean ore and secondary land use (i.e. agriculture) might have triggered colluvial deposition on the Swabian Jura [96,97]. The overall impression is a decline of used land during the Iron Age because there were very few data from the southeastern Black Forest and the Baar area. The exceptions are the Fuerstenberg and Spaichingen sites. In Spaichingen, located at the lower slope of Swabian Jura cuesta, there are four ^14^C and four OSL ages, indicating a major phase of land use at this site, which correlates with archaeological findings [37]. This local phase of increased colluviation on the Baar and the Swabian Jura (*ca* 500 BCE [19]) falls in a cold period [90,93,98]. The rather unfavourable climate in addition to low land use intensity [99] and population density [100] might have resulted in the formation of spatially different intensities of colluvial deposition.

#### 4.1.5. Roman empire

In contrast to the expected intensification of land use and soil erosion, land use-related soil erosion seems to have declined towards the Roman Empire. Only two Roman charcoal fragments and one OSL age date to this period at the Swabian Jura sites, indicating a generally low land use intensity. Samples from Lin2 complement the archaeological evidence of Roman settlements. Even though there are known Roman settlements on the Baar, this is not visible in colluvial deposits. The Spaichingen site is located near a formerly productive spring and next to a Roman road connecting Tuttlingen and Rottweil north of Spaichingen [101], but only one age falls into the period of the Roman Empire. Charcoal fragments dated to the Roman Empire found in Grueningen indicate increased human activities or colluvial deposition [19], which is the only main colluviation phase falling into a dry and warm period [93,98]. The triggering activity might have been practising agriculture on the fields to support a Roman castrum a few kilometres south near Huefingen, which supposedly accommodated about 1000 soldiers [102,103]. The wood of the Black Forest might have been used by the Romans [104], but these activities did not lead to an increase of colluvial deposition in the Black Forest. It can be interpreted that the selected logging sites were not intensively used by the Romans.

#### 4.1.6. Middle ages

Land use and settlements are archaeologically well documented on the Swabian Jura during the Middle Ages. The high to late medieval climate was warm until the Little Ice Age and the transition to the early Modern period [93,98,105]. The forests were cut purposefully to use the wood or to clear areas for farming or animal husbandry, having left less than 20% of the forest cover during that period [99,106,107]. A striking situation is the limited archaeopedological record on the Swabian Jura, with only two sites dating to the late High Middle Ages. However, considering the entire study area, especially the Baar area, the increased colluviation and land use become apparent. One explanation for the low record of colluvial deposition on the Swabian Jura could be that these sites were not particularly used for agriculture. Another point could be the potential incorporation of medieval colluvial deposits into the modern Ap horizon.

### 4.2. Geomorphodynamically stable times—dating the gaps of colluvial deposition

The reconstruction of phases of colluvial deposition allows us in return to infer phases of relative geomorphodynamic stability and the formation of colluvial deposits, without soil erosion (figure 1). These phases are the deposition gaps between colluvial layers. The duration of these stable periods is the difference between the ages of the colluvial horizons. The underlying older horizon would have served as a land surface during that time and, therefore, it was hypothesized that it might show different pedogenic properties (such as an enrichment in SOC or heavy metals).

To reconstruct geomorphodynamically stable times, we used all available data from the project (see supplementary data and [19,26]). For example, the upper 88 cm of colluvial material in soil profile Bri1 [26] is not distinguishable by dating and was deposited during Modern times. The underlying colluvial horizon dates to the Roman Empire and Merovingian period, and can thus be interpreted as having served as a land surface at some point during the High and Late Middle Ages. The environmental conditions during those roughly 1000–1500 years might have been geomorphodynamically stable with little to no or sustainable land use, or it might have been an active land surface with deposition and erosion, which might have eroded the possibly medieval soil. The dated charcoals predate the OSL ages of the respective colluvial horizons, but they also indicate a time gap at a depth of 88 cm. An enrichment of soil chemicals would point to the soil horizon being used as a land surface; however, no such enrichment could be shown for the measured elements in the depth of 88–93 cm. Instead, the lower part of the covering colluvial horizon is enriched in SOC, Ni, Pb, Cr and clay [26]. This can be interpreted as relicts of former land use and a reworking of the used former land surface into the modern soil horizon.

Leh3 also has a depositional gap between the lowermost colluvial horizon (2M3), dating to the Neolithic to Bronze Age transition, and the covering (M2) colluvial horizon at a depth of 51 cm, dating to Modern times. The 2M3 horizon was, therefore, the land surface for about 3600–4500 years. Charcoal ages indicate an even longer land use/deposition gap of about 5500–6000 years between the Late Neolithic and the High Middle Ages. However, soil chemical analyses show only very slight enrichment of SOC in the upper part of 2M3 and similar contents of Cr, Ni, Pb and clay in the lower part of M2 and the upper part of 2M3. Human effects on pedogenic properties seem to be weak given the long duration of potential land use.

The profile Spa4 shows three packages of colluvial deposits, the oldest of which developed during the Middle Bronze Age to the Urnfield period (3M6) and was the land surface for about 200–1500 years until the covering colluvial deposits were formed during the Iron Age and Roman times (3M5, 3M4, 3M3). Roman deposits might have been the land surface for 500–1000 years until they were covered by medieval deposits (2M2, M1). The boundary between the medieval and Roman-time deposits at a depth of 88 cm has differences in properties, i.e. an increase of Ni, clay and SOC above the boundary and an increase of Cu below 88 cm.

Profile Lin2 contains two potential former land surfaces, the lower one at around a depth of 60 cm for 800–1700 years during the Iron Age and the upper boundary between colluvial layers at 35 cm for 1000–1500 years during the Middle Ages. Except for the different ages of colluvial horizons, the soil shows no differences between the properties of ‘neighbouring’ colluvial horizons.

The hypothesis of increased SOC contents in the upper part of a potential former land surface as a result of elevated input by plants has to be declined. The comparison (table 2) shows that the SOC content of the upper 5 cm of the lower colluvial horizon on average is slightly lower (−0.09%) than that of the above lying colluvial horizon. Considering the complete colluvial horizon, the difference is more pronounced (−0.39%). There are no correlations of SOC content differences with the time period or the length of the time gap; the latter can be understood as the duration of potential land use. It can be concluded that if a lower colluvial horizon was a former land surface, the upper part with the Ah horizon (enriched in SOC) must have been reworked into the overlying colluvial horizon and/or transported downslope by erosion. This might also explain the tendency of higher SOC contents in the lower part of the colluvial horizons. The overall pattern of declining SOC contents with increasing depth has to be kept in mind.

**Table 2.** Comparison of SOC contents between soil horizons separated by a potential former land surface. Positive differences indicate a higher SOC content in the upper sample. Time gap refers to the difference between the relevant OSL ages.

site	depth (cm)	upper horizon	lower horizon	SOC difference*^a^* (%)	SOC difference*^b^* (%)	time gap (years)	period of the potential land surface
Rus3	46	M2	2M3	−0.30	−1.30	1270–1680	Middle Ages
Rus3	105	2M3	2M4	0.03	−0.35	610–800	Latène period–Roman
Lin2	35	M2	2M3	0.01	−0.12	1070–1550	Middle Ages
Lin2	60	2M3	3M4	−0.05	−0.61	820–1660	Iron Age
Spa4	88	2M2	3M3	0.00	0.04	440–1040	Roman–Merovingian
Spa4	195	3M5	3M6	0.02	0.23	230–1330	Hallstatt period
Spa1	145	M4	2M5	−0.17	−0.09	800–1400	Roman–Merovingian
Mag1_14	60	M2	M3	−0.39	−0.68	700–3500	Middle Ages–Late Neolithic
Leh3	51	M2	2M3	0.31	−0.57	3600–4480	Middle Ages–Middle Bronze Age
Bri1	88	3BgM1	4BgM2	−0.32	−0.48	960–1440	Roman–Merovingian

*^a^*SOC difference between immediate sampled depth increments (subsamples of neighbouring soil horizons).

*^b^*SOC difference of the two ‘neighbouring’ soil horizons.

SOC/N ratios, in contrast, are slightly higher (0.24%) in the upper 5 cm of colluvial horizons and are generally higher (0.42%) in the lower colluvial horizon, compared to the directly covering colluvial horizon. This comes from lower N contents in the lower parts or it may picture the generally higher rate of N content decline with depth compared to the depth function of the SOC content across the whole soil profile. The higher C/N ratios might depict the influence of the former land surface.

## 5. Conclusion

Analysis of archaeopedological age determinations suggest that land use leading to local long-lasting effects on the landscape began during the Mesolithic, but OSL ages and OSL-SPDs suggest that only during the Neolithic intensified land use resulted in colluvial deposition on certain sites. However, widespread colluvial deposits appeared only during the Urnfield period and Iron Age, suggesting a significant human impact on the landscape during these times. Major phases of colluvial deposition also occur during the High Middle Ages and the Early Modern period, perhaps indicating unsustainable land use. But what drove land use changes in the past? One oft-cited cause of settlement and land use change is the climate [25]. For example, a warming climate might make territory at higher altitudes available to settlers. Following this climate-forcing hypothesis in SW Germany, it would suggest that the Black Forest and the Swabian Jura should have been settled later than was the Baar, and only when the climate was warmer. Climate fluctuations are documented within the Holocene [108] and might be helpful to explain settlement dynamics. The favourable Baar area was indeed settled and used earlier than the western Swabian Jura and the southeastern Black Forest, but those two unfavourable areas are also different from each other. Blümel [106] argues that a certain stability and, therefore, predictability of ideally favourable climate conditions can encourage the development of cities and trade, because they benefit from a reliable surplus production in the surrounding areas and food supply for the city population [106]. Following this supposition, favourable climatic conditions can be understood as stable and foreseeable conditions without sudden changes and within certain limits of temperature and precipitation [108].

However, the spatio-temporal distribution of the colluvial deposits analysed in this study cannot only be explained by varying environmental conditions. Soil erosion and accumulation of colluvial deposits are dependent on precipitation, which explains the correlation with time periods with higher precipitation and lower temperatures. Particularly temperature, precipitation, relief, natural resources and soil are environmental variables, which can be understood as framework conditions, needed to be within certain limits to allow settlements and land use. Cultural variables such as trading, religion, state evasion, conflicts, technical progress and population pressure seem to explain the detailed form of land use dynamics.

Raw materials (such as ore, wood, rock, salt) have always been available in the Black Forest and Swabian Jura but were not used at all points in time, which indicates that cultural conditions might have played a crucial role in the distribution of settlements and used land. Such cultural conditions might have been the rise of the population density, which led to an intensification of land use as it is shown for the beginning of the Neolithic (figure 10; [72]). Another explanation is the state of technological development, which allowed people to use raw materials in a new way. Religious practices, trade or communication might also influence the use of raw materials as resources. The economic, cultural, political or social need for resources in favourable areas might explain the use of nearby unfavourable (in terms of soil and climate) areas such as low mountain ranges of the Black Forest and the Swabian Jura. Other explanations might be to evade state interference by inhabiting marginal areas [109] or the retreat to secluded areas for religious reasons. Once these unfavourable areas were occupied, social dynamics come into play and changing environmental conditions do not necessarily lead to the abandonment. Diversification of land use, for example, might have made it possible to continuously use unfavourable areas, sustained by the buffering capacity and resilience of societies [110,111].

The analysis of colluvial deposits reported in this study contributes to national and international efforts to protect soils as a natural non-renewable resource. In some cases, these soils also act as archives of past human activities which can provide insights into societal and environmental sustainability. It is also necessary to know about the past to assess and think about recent activities and possible future (un)intended outcomes. Colluvial deposits combine all three topics and are thus ideal research objects to reconstruct past land use change and the human–environment relation.

## Data accessibility

The data used in this paper are stored at Dryad: (http://dx.doi.org/10.5061/dryad.rh67h) [75]. These are Excel spreadsheets (.xlsx) and .csv files which contain the soil descriptions, soil laboratory results, and AMS-^14^C and OSL dating results. Additional information and supplementary data about this project are published [19,26]. This publication is part of the dissertation by J.H., which will be available online (https://publikationen.uni-tuebingen.de/xmlui/handle/10900/42126).
